# 

**Published:** 1858-12

**Authors:** 


### THE DENTAL REGISTER OF THE WEST.

EDITED BY

J. TAFT AND GEO. WATT.

December, 1858.

Vol 12 — 2

Published Quarterly at $3 Per Annum.

CINCINNATI:
J. TAFT, PUBLISHER AND PROPRIETOR.
1858.

